# Association between Big Five personality factors and medication adherence in the elderly

**DOI:** 10.47626/2237-6089-2020-0143

**Published:** 2022-06-14

**Authors:** Natascha Melo Linkievicz, Vanessa Sgnaolin, Paula Engroff, Gabriel Behr Gomes Jardim, Alfredo Cataldo

**Affiliations:** 1 Programa de Pós-Graduação em Gerontologia Biomédica PUCRS Porto Alegre RS Brazil Programa de Pós-Graduação em Gerontologia Biomédica, Pontifícia Universidade Católica do Rio Grande do Sul (PUCRS), Porto Alegre, RS, Brazil.; 2 Instituto de Geriatria e Gerontologia PUCRS Porto Alegre RS Brazil Instituto de Geriatria e Gerontologia, PUCRS, Porto Alegre, RS, Brazil.; 3 Service Universitaire de Psychiatrie de l’Age Avance University of Lausanne Lausanne Switzerland Service Universitaire de Psychiatrie de l’Age Avance, University of Lausanne, Lausanne, Switzerland.; 4 Escola de Medicina PUCRS Porto Alegre RS Brazil Escola de Medicina, PUCRS, Porto Alegre, RS, Brazil.

**Keywords:** Aged, medication adherence, personality

## Abstract

**Introduction:**

Adherence to medications can be associated with circumstances related to the patient, with the pathology, with cultural health beliefs, with habits, and with quality of life. Behavioral patterns can also directly influence a patient’s pharmacological adherence, since they are related to their perception and understanding of their own health status and of their drug and non-drug treatments.

**Objective:**

To investigate the association between adherence to pharmacological treatment and personality factors, sociodemographic variables, and economic data in the elderly.

**Methods:**

Cross-sectional descriptive study. The population studied were elderly people registered with the Family Health Strategy of Porto Alegre and enrolled on the Brain Aging Program (PENCE), from March 2013 to November 2015. Sociodemographic data, pharmacological adherence, and personality traits were evaluated. Exclusion criteria were incomplete data in the personality and pharmacological adherence assessments; cognitive impairment, evaluated using the instrument Mini-Mental State Examination (MMSE), or not having carried out this assessment.

**Results:**

A total of 123 individuals were included with a mean age of 71.35±7.33 years, 58.6% of whom reported some level of non-adherence to their medication regime (low and moderate adherence). Elderly people with low adherence had significantly higher mean scores in the Neuroticism factor, while those with high adherence had significantly higher mean scores in the Agreeableness and Conscientiousness factors.

**Conclusion:**

The study suggests that pharmacological adherence among the elderly is negatively associated with the Neuroticism personality trait, while the Agreeableness and Conscientiousness traits are positively associated.

## Introduction

In Brazil, 85% of the elderly population have at least one chronic disease, and approximately 15% have multiple comorbidities, such as hypertension, diabetes, and cardiovascular problems.^1^ This scenario results in the elderly using greater numbers of different medications, making the practice of polypharmacy common.^2^ Use of various medications combined with therapeutic complexity can make adherence to treatment more difficult. High rates of non-adherence, which can exceed 80%, have been observed in the elderly when compared to the general population.^3^ This non-adherence can have a detrimental influence on patient health status and may generate other complications, such as a reduced quality of life, greater risk of hospitalization, and increased health care costs.^1,3^

Adherence to medications is associated with several circumstances related to the patient (cognitive function, impaired sight, lack of understanding, inability to administer different medications), with the pathology, with cultural health beliefs, with habits, and with quality of life, as well as with the relationship with the healthcare team.^4^ It is important to point out that behavioral patterns can also directly influence patients’ pharmacological adherence, as they are related to their perception and understanding of their own health status and drug and non-drug therapies.^3,5^ Personality traits are enduring behavioral patterns that tend to be stable throughout life. They set the standards of behavior and interpersonal relationships.^6^ Therefore, personality traits can also influence adherence behaviour.^7^

According to the Five Factors Model (Big Five), people’s personality traits can be defined as predispositions to behave in a certain way and can be grouped into five major dimensions: Neuroticism, Extraversion, Openness to Experience, Agreeableness, and Conscientiousness.^8^ Some studies have linked personality dimension scores to the degree of patients’ adherence to their respective treatments, showing that individuals with chronic diseases had a negative response to adherence when classified with high levels of Neuroticism, while Agreeableness and Conscientiousness were positively correlated with adherence.^9^ The Neuroticism factor was also associated with poorer adherence to the medication program in elderly patients undergoing preventive treatment for dementia.^10^

In order to improve therapeutic adherence, interventions could take personality traits and the reasons for adherence being less than ideal into consideration.^7^ Therefore, the present study aimed to investigate associations between adherence to pharmacological treatment and five-factor model personality traits, sociodemographic variables, and economic data in the elderly.

## Methods

### Study design

The study design is cross-sectional and descriptive and this study is part of the umbrella project entitled the Brain Aging Program (PENCE), a partnership between the Pontifical Catholic University of Rio Grande do Sul (PUCRS) and the Municipal Health Secretariat of Porto Alegre. The present study is a subproject and was supported by the Visiting Professor Abroad Program (PVE), a collaboration between the University of Lausanne Psychiatry Service (Switzerland) and the PUCRS Institute of Geriatrics and Gerontology (Porto Alegre, Brazil).

### Population and sample studied

The sample population was formed of elderly individuals registered with the Family Health Strategy (FHS) at the PUCRS São Lucas Hospital who were part of the PVE subproject. The inclusion criteria were 60+ years of age; registered with PENCE; taking at least one medication; and agreeing to participate in the research by signature of an Informed Consent Form (ICF). Exclusion criteria were incomplete data in the personality and medication adherence assessments; cognitive impairment, as assessed by the Mini-Mental State Examination instrument (MMSE); or not having undertaken this assessment.

### Data collection

Data collection took place from March 2013 to November 2015. The first stage of the PENCE program involved training the FHS teams, after which community health agents (CHA) registered the elderly individuals linked to their FHS, administering a general questionnaire to collect sociodemographic, lifestyle, pharmacological, and health data, as well as applying screening tools for cognition and depression. Elderly individuals who had disorders according to these instruments were subsequently referred to the Cerebral Aging Clinic (AMBEC), São Lucas Hospital, PUCRS, for further medical evaluation.

A subsample of the elderly people who attended the AMBEC were selected for the PVE. Several tests were applied in addition to the medical evaluation, but for the purposes of the present study the MMSE and the NEO-Five Factor Inventory (NEO-FFI) instruments were selected, since they complement the objective of the study.

The elderly participants’ cognitive function was assessed using the MMSE screening instrument. The evaluator marked one point for each correct answer, with a maximum score of 30 points.^11^ Cutoff points were set according to the educational level of the individual, with 13 points for illiterate participants, 18 points for individuals with a low or medium level of education, and 26 points for those with a high level of education.^12^

The NEO-FFI instrument was used to assess personality traits.^13^ This instrument is a short version of the Revised NEO Personality Inventory, which has been translated into and validated for Portuguese.^14^ The questionnaire has 60 items and assesses the five main dimensions: Neuroticism (emotionally unstable, susceptible to psychological stress, maladjustment and negative emotionality); Extraversion (energetic, assertive); Openness to Experience (imaginative, independent mind and intellectual curiosity); Conscientiousness (responsible, reliable, orderly); and Agreeableness (empathetic, cooperative). The instrument has a five-point Likert response format, ranging from strongly disagree to strongly agree.^15^

The Morisky Scale^16^ was used to evaluate adherence to medication treatment by the elderly participants. It was inserted into the PENCE general questionnaire, which was applied by the CHA. The instrument consisted of the following questions: 1) Do you sometimes forget to take your medications? 2) Are you careless with the times for taking your medications? 3) When you are feeling better, do you sometimes stop taking your medications? 4) At some point, if you feel bad, do you stop taking your medications? Each yes answer received a point. Thus, an elderly person who scored no points was considered to have high adherence to pharmacological treatment; one to two points was classified as moderate adherence; and three to four points, as low adherence.

### Statistical analysis

Data analysis was performed using the Statistical Package for the Social Sciences (SPSS), version 17. Variables were described using frequencies, means, and standard deviations. Associations between categorical variables were tested using Pearson’s chi-square test and, in specific cases, the chi-square test for linear trend. One-way analysis of variance (ANOVA) was used to compare means, followed by the Tukey post-hoc test. A 95% confidence interval was also established to demonstrate a statistically significant difference between the groups analyzed. Results were considered significant when p < 0.05.

### Ethical considerations

The research project was approved by the PUCRS Research Ethics Committees (nº 826,858) and by the Porto Alegre Municipal Health Secretariat (nº 1,003,962). All participants or their legal representatives provided signed informed consent.

## Results

A total of 123 individuals were included in the study, with a mean age of 71.35±7.33 years (range 60-91 years), majorities of whom were women, had low educational level, were married, had no caregiver, and had a family income of 1 to 3 times the minimum salary. In relation to pharmacological adherence, 58.6% of the elderly people included in the study reported some level of non-adherence to their medication regimens (low and moderate adherence). No statistical differences were observed when evaluating associations between adherence and sociodemographic or economic data (Table 1).

**Table 1:** Morisky Scale assessment by sociodemographic and economic data

Variables	Total	Adherence (Morisky)			P
		Low	Moderate	High	
Gender					
Female	98 (79.7)	15 (15.3)	47 (48.0)	36 (36.7)	0.102
Male	25 (20.3)	3 (12.0)	7 (28.0)	15 (60.0)	
Age group (years)					
60-69	61 (49.6)	12 (19.7)	23 (37.7)	26 (42.6)	0.543*
70+	62 (50.4)	6 (9.7)	31 (50.0)	25 (40.3)	
Schooling (years of study)					
0	41 (34.2)	7 (17.1)	18 (43.9)	16 (39.0)	0.853*
1-4	35 (29.2)	4 (11.4)	17 (48.6)	14 (40.0)	
5-8	30 (25.0)	3 (10.0)	13 (10.8)	14 (46.7)	
9+	14 (11.6)	4 (28.6)	4 (28.6)	6 (42.8)	
Marital status					
Single	23 (19.2)	6 (26.1)	7 (30.4)	10 (43.5)	0.463
Married	49 (40.8)	5 (10.2)	26 (53.1)	18 (36.7)	
Divorced	9 (7.5)	2 (22.2)	4 (44.4)	3 (33.3)	
Widowed	39 (32.5)	5 (12.8)	16 (41.0)	18 (46.2)	
Has caregiver					
Yes	26 (21.8)	3 (11.5)	11 (42.3)	12 (46.2)	0.696
No	93 (78.2)	15 (16.1)	43 (46.2)	35 (37.6)	
Family income (minimum salary)					
Up to 1x	27 (24.5)	4 (14.8)	9 (33.3)	14 (51.9)	0.968*
1-3x	66 (60.0)	10 (15.2)	32 (48.5)	24 (36.4)	
3-10x	17 (15.5)	1 (5.8)	8 (47.1)	8 (47.1)	
					
Total	123 (100)	18 (14.6)	54 (43.9)	51 (41.5)	

Data presented as n (%).

The totals for some variables may not attain the overall n of 123 due to sample losses.

* Linear chi-square test.

The results for pharmacological adherence and personality traits were also analyzed, demonstrating that elderly people with low adherence had significantly higher mean scores for the Neuroticism factor, mainly when compared with the moderate subset (p = 0.015). While those with high adherence had significantly higher mean scores for Agreeableness, especially when compared to the low subset (p = 0.024), and for Conscientiousness, in the comparisons high versus low adherence subsets (p = 0.016) and also moderate versus low subsets (p = 0.044) (Table 2).

**Table 2:** Morisky Scale assessment by personality traits (NEO-Five Factor Inventory)

	Adherence (Morisky)			Adherence (Morisky)			p
	Low	Moderate	High	Low	Moderate	High	
	Mean ± SD	Mean ± SD	Mean ± SD	Mean ± SD	Mean ± SD	Mean ± SD	
Neuroticism	43.44±11.93	35.78±10.00	37.44±9.19	37.51-49.38	33.05-38.51	34.83-40.05	0.021
Extraversion	34.83±10.33	38.19±6.64	37.63±7.45	29.70-39.97	36.37-40.00	35.53-39.72	0.267
Openness to experience	33.39±6.72	35.61±4.07	34.71±4.24	30.05-36.73	34.50-36.72	33.51-35.90	0.195
Agreeableness	37.44±7.72	40.30±3.61	40.80±4.08	33.61-41.28	39.31-41.28	39.66-41.95	0.029
Conscientiousness	41.67±8.69	45.37±5.31	45.98±4.47	37.34-45.99	43.92-46.82	44.72-47.24	0.020

Data presented as mean ± standard deviation.

SD = standard deviation; 95%CI = 95% confidence interval.

## Discussion

The present study showed that most of the elderly participants were non-adherent to their drug treatments. The literature similarly demonstrates that the elderly population has high rates of non-adherence. A study in Salto Grande (São Paulo, Brazil) revealed that 85.3% of elderly people did not adequately adhere to their prescribed drug treatments.^17^ A study in Cuiabá (Mato Grosso, Brazil) found 80% of low adherence among the elderly.^5^ Low adherence among geriatric patients is due to emergence of multiple chronic diseases developed over the course of the aging process, which make polypharmacy and self-medication practices more likely. These are associated with adverse reactions and pharmacological interactions.^1,18^

Other factors are also related to low adherence to medications in the elderly, such as the complexity of administering dosing schedules, difficulty in swallowing, denial or fear of illnesses, forgetfulness problems, low self-esteem, interruption of treatment for alcohol consumption, low economic income, and educational level.^1,4,18^ The literature shows that individuals with worse socioeconomic levels, represented by low income and low educational levels, have lower adherence to pharmacotherapy.^19^ This is due to possible difficulties in understanding the treatment protocols and health guidelines, in addition to financial problems, which may interfere with access to medicines.^19^ In the present study, this association was not evidenced due to the homogeneous profile of the population, which was composed of elderly people with low levels of income and education, hindering reliable comparison between individuals of different socioeconomic levels.

When personality factors were taken into consideration, the study indicated that elderly people with low adherence have significantly higher mean scores for Neuroticism, corroborating findings in the literature that associate this trait with a series of unfavorable results in relation to adherence to treatments and health behaviors.^9,10,20,21^ In a study by Axelsson et al., 5,000 30-to-70-year-old residents of Sweden were randomly invited to participate in a cross-sectional study of the influence of personality traits on adherence. Based on this sample, 749 individuals had a chronic disease and were selected for evaluation. The results showed that patients with chronic diseases and high Neuroticism scores were less adherent to their treatment.^9^ Jerant et al. investigated the relationship between personality factors and adherence to dementia prevention treatment. A total of 771 elderly people aged 72 years or over and enrolled on the Ginkgo Evaluation of Memory study were evaluated. Participants were followed for six years and the results indicated an association between the Neuroticism factor and non-adherence by the elderly participants to their medication programs.^10^

Neuroticism refers to the way individuals deal with their negative emotions and behave when faced with complicated situations. People with high scores in this trait are more anxious, concerned, and have different ideas of reality.^10,22^ Thus, they have greater difficulty in coping with frustrations in response to situations and certain stimuli that other individuals would easily be able to face.^10^ Neuroticism is also linked to development of physical and pathological disorders and eating disorders and is considered a predictor of smoking and relapse in alcoholics.^22^

Multiprofessional health teams must be trained to identify and help these individuals, knowing how to recognize early signs of excessive distress due to the complexity of treatments, their side effects and economic difficulties. Therefore, it is important to intervene with strategies that promote medication adherence, for example, extending the duration of multidisciplinary care, improving communication and bonding with the patient, and better clarifying treatment information through illustrative written and audiovisual materials.^5,10,17^ It is essential that patients feel welcomed and are willing to correctly follow the pharmacotherapy prescribed. In this sense, pharmaceutical care can be an effective strategy to guarantee these interventions.^5,17^

The elderly participants classified as having high adherence presented higher mean scores in the dimensions Agreeableness and Conscientiousness, confirming that the higher the scores for these factors, the greater these individuals’ therapeutic adherence.^9,20^ Axelsson selected 445 individuals who reported using antibiotics in a study that explored the relationship between personality traits and adherence to antibiotic therapy. The study concluded that Agreeableness and Conscientiousness positively influence adherence behavior.^20^ In two recent surveys, one with older adults from Taiwan and the other with octogenarians and centenarians from Georgia, it was observed that Agreeableness and Conscientiousness are also strongly associated with the conditions that determine successful aging.^21,23^ Agreeableness originates socially pleasant behaviors and individuals with high scores in this factor demonstrate characteristics such as generosity, trust, and engagement.^22^ These individuals are prone to being understanding and cooperative.^20^ Conscientiousness is linked to impulse control and performance of obligations and duties. People with high levels of this factor have characteristics such as caution, organization, persistence, responsibility, and trust.^22^ They tend to have high cognitive ability, a capacity to easily interact socially, and conduct prudent health behaviors, such as maintaining a routine, having hobbies, and taking better care of their physical and psychological problems.^21,23^

Taking into consideration these personality characteristics, we can understand that the results reinforce how personality traits are associated with elderly people’s perceptions with regard to medications and, consequently, with treatment adherence. For example, patients who believe the prescribed treatment is necessary for their health are more inclined to adhere to drug therapy. However, individuals who are concerned about the possible side effects that a drug may cause are more likely not to correctly adhere to pharmacotherapy.^24^

This study has some limitations, such as the scarcity of literature involving the relationship between personality traits and medication adherence in the elderly population. Most of the studies already carried out consider other age groups, making it difficult to compare results and validate evidence. There are also limitations resulting from the cross-sectional design, such as the impossibility of establishing the temporal relationship between associated factors. The results apply to the population registered with the FHS, which is focused on primary healthcare and, therefore, may not be representative of the entire Brazilian population. The objective of this study was not to evaluate clinical variables such as number of medications used, duration of medical treatment, type of disease, or treatment received, which could also be considered a study limitation.

## Conclusion

The present study suggests that adherence to pharmacological treatment in the elderly is negatively associated with the Neuroticism personality trait, while the Agreeableness and Conscientiousness traits have positive associations. The results emphasize the importance of routinely investigating personality factors in the elderly during outpatient follow-up appointments, given they are more likely to be subject to polypharmacy and, consequently, more susceptible to non-adherence to their medical prescriptions.

Investigation of the Big Five personality traits can assist the multi-professional team to create strategies taking account of these traits and adjust treatment according to an individual’s behavioral characteristics, thereby improving medication adherence and treatment success. These measures can help reduce health service costs and the negative effects of non-adherence on comorbidities, providing better health and quality of life for the elderly.
